# Biochemical and immunological characterization of an ETEC CFA/I adhesin cholera toxin B subunit chimera

**DOI:** 10.1371/journal.pone.0230138

**Published:** 2020-03-16

**Authors:** Michael G. Jobling, Steven T. Poole, Fatima Rasulova-Lewis, Aisling O’Dowd, Annette L. McVeigh, Amit Balakrishnan, Stephanie A. Sincock, Michael G. Prouty, Randall K. Holmes, Stephen J. Savarino

**Affiliations:** 1 Department of Immunology and Microbiology, University of Colorado School of Medicine, Aurora, CO, United States of America; 2 Henry M. Jackson Foundation for the Advancement of Military Medicine, Bethesda, MD, United States of America; 3 Enteric Diseases Department, Naval Medical Research Center, Silver Spring, MD, United States of America; 4 Department of Pediatrics, Uniformed Services University of the Health Sciences, Bethesda, MD, United States of America; Instituto Butantan, BRAZIL

## Abstract

Surface-expressed colonization factors and their subunits are promising candidates for inclusion into a multivalent vaccine targeting enterotoxigenic *Escherichia coli* (ETEC), a leading cause of acute bacterial diarrhea in developing regions. However, soluble antigens are often poorly immunogenic in the absence of an adjuvant. We show here that the serum immune response to CfaE, the adhesin of the ETEC colonization factor CFA/I, can be enhanced in BALB/c mice by immunization with a chimeric antigen containing CfaE and pentameric cholera toxin B subunit (CTB) of cholera toxin from *Vibrio cholerae*. We constructed this antigen by replacing the coding sequence for the A1 domain of the cholera toxin A subunit (CTA) with the sequence of donor strand complemented CfaE (dscCfaE) within the cholera toxin operon, resulting in a dscCfaE-CTA2 fusion. After expression, via non-covalent interactions between CTA2 and CTB, the fusion and CTB polypeptides assemble into a complex containing a single dscCfaE-CTA2 protein bound to pentameric CTB (dscCfaE-CTA2/CTB). This holotoxin-like chimera retained the GM1 ganglioside binding activity of CTB, as well as the ability of CfaE to mediate the agglutination of bovine red blood cells when adsorbed to polystyrene beads. When administered intranasally to mice, the presence of CTB in the chimera significantly increased the serum immune response to CfaE compared to dscCfaE alone, stimulating a response similar to that obtained with a matched admixture of dscCfaE and CTB. However, by the orogastric route, immunization with the chimera elicited a superior functional immune response compared to an equivalent admixture of dscCfaE and CTB, supporting further investigation of the chimera as an ETEC vaccine candidate.

## Introduction

Enterotoxigenic *Escherichia coli* (ETEC) is a leading cause of acute bacterial diarrhea in young children in and travelers to developing countries [1–7]. ETEC adherence to the small intestine is promoted by the expression of one or more colonization factors (CFs), of which over 25 have been described [8]. After colonization, ETEC elicits fluid and electrolyte secretion through the expression of heat-labile (LT) and/or heat-stable (ST) enterotoxins [9]. The importance of CFs and toxins in ETEC pathogenicity make them appealing targets for ETEC vaccine strategies. Given that ETEC express numerous serologically distinct CFs, the development of a broadly-protective ETEC vaccine is challenging, requiring a multivalent approach containing a subset of epidemiologically prevalent CFs, as well as a form of LT toxoid [10, 11].

CFA/I, the archetype of Class 5 ETEC fimbriae and a commonly expressed ETEC CF, is comprised of two subunits, multiple copies of CfaB, which forms the fimbrial stalk, and a tip-localized adhesin, CfaE [12]. It has been demonstrated that CfaE orchestrates the CFA/I-mediated agglutination of bovine and human erythrocytes in an *in vitro* mannose-resistant hemagglutination assay (MRHA), as well as binding to human intestinal epithelial cells *in vitro* [13, 14]. Additionally, parenteral immunization of rabbits with a donor-strand complemented form of CfaE, dscCfaE, and incomplete Freund’s adjuvant generated a functional immune response against ETEC strain H10407 (CFA/I^+^), inhibiting the ability of the strain to induce hemagglutination [13]. In both mice and the new world monkey *Aotus nancymaae*, dscCfaE is immunogenic when co-administered intranasally with the genetically attenuated heat-labile enterotoxin LTR192G [15] and protects against challenge with the H10407 ETEC strain (CFA/I^+^) in both the infant mouse and the *A*. *nancymaae* challenge models [16, 17]. Importantly, bovine colostral anti-CfaE IgG antibodies provided protection in humans against CFA/I+ ETEC challenge [18], supporting continued evaluation of CfaE as a vaccine candidate. Although dscCfaE is immunogenic alone, we have shown that its immunogenicity is enhanced when co-administered with an adjuvant [15, 17].

Cholera toxin (CT) from *Vibrio cholerae* has been used as an adjuvant to boost mucosal and systemic responses to weakly immunogenic antigens [19–25]. CT consists of an A subunit (CTA) and a pentameric B subunit (CTB) associated noncovalently. CTB harbors the GM1 ganglioside binding activity, which allows CT to bind and enter host intestinal epithelial cells. CTA contains two domains, CTA1 and CTA2. CTA1 is associated with the toxicity of CT and CTA2 interacts with CTB, allowing for the assembly of the CT holotoxin. However, incorporation of CT into a clinical vaccine formulation is challenging since the holotoxin is diarrheagenic when given orally in doses greater than 2.5 μg and LT, a close relative to CT, was associated with Bell’s palsy when used an adjuvant for intranasally delivered influenza vaccine [26, 27]. CTB lacks the diarrheagenic activity of the CTA1 domain and may be a safer alternative to CT holotoxin, especially for oral administration. Like CT, CTB has been shown to adjuvant immune responses to a co-administered antigen, including CfaE [15, 28]. Furthermore, incorporation of CTB into a vaccine formulation would likely enhance efficacy of an ETEC vaccine given that immunization with a cholera vaccine containing CTB has shown short-term efficacy against LT-ETEC mediated diarrhea due to the cross-protective immune response elicited by CTB [29].

We previously described the generation of holotoxin-like chimeras by genetic replacement of the CTA1 domain of CT with exogenous proteins. The resulting chimeric proteins, retained the ability to bind to GM1, lacked the toxicity of native CT, and preserved the functional conformation of the substituted protein [30]. This strategy of CTA1-substitution has been successfully used in the production of CT-like chimeras containing LcrV antigen from *Yersinia pestis*, pertussis toxin from *Bordetella pertussis*, SREHP from *Entamoeba histolytica*, MrpH of *Proteus* mirabilis, and TcpA or TcpF from *Vibrio cholerae*. Immunization with these CT-like chimeras led to increases in the mucosal immune responses to the incorporated antigens [31–36].

Herein, we describe the construction and characterization of a dscCfaE-CTA2/CTB complex (chimera), a CT-like protein in which CTA1 has been replaced by dscCfaE. Additionally, using a well-established murine vaccination model [15], we evaluated whether the chimera could generate a robust, functional anti-CfaE serum response in the absence of an exogenous adjuvant.

## Materials and methods

### Bacterial strains, plasmids and primers

TE1 [F′ lacI^q^ Tn*10* (TcR), *glnV*44 Δ*endA*] [37] was used for routine cloning and initial expression screening. BL21 (Novagen, Madison, WI) was used for protein expression. Cells were made competent with Z-competent buffers (Zymo Research, Corp., Orange, CA). Bacteria were grown in LB with 25 ug/mL chloramphenicol (Cm). Cloning vectors used were pARLDR19 [36], which is based on the pAR3 vector [38]. Standard cloning protocols [39] were used for polymerase chain reactions (PCR) using Pfu polymerase, DNA restriction digestion, and ligations. DNA fragments were purified from agarose gels using UltraClean resin (MoBio labs, Carlsbad, CA). Primers to amplify the gene for donor strand complemented CfaE (dscCfaE) were CFaEdscSphF (5’ GCCGTGCATGCAGATAAAAATCCCGG 3’) and CFaEdscClaR (5’ GCTATCGATTGCAAAAGATCAATCAC 3’)–added restriction sites underlined.

### Construction of expression vectors for dscCfaE-CTA2/CTB (p0809C304) and CTB (p0826C1)

**Fig 1:**
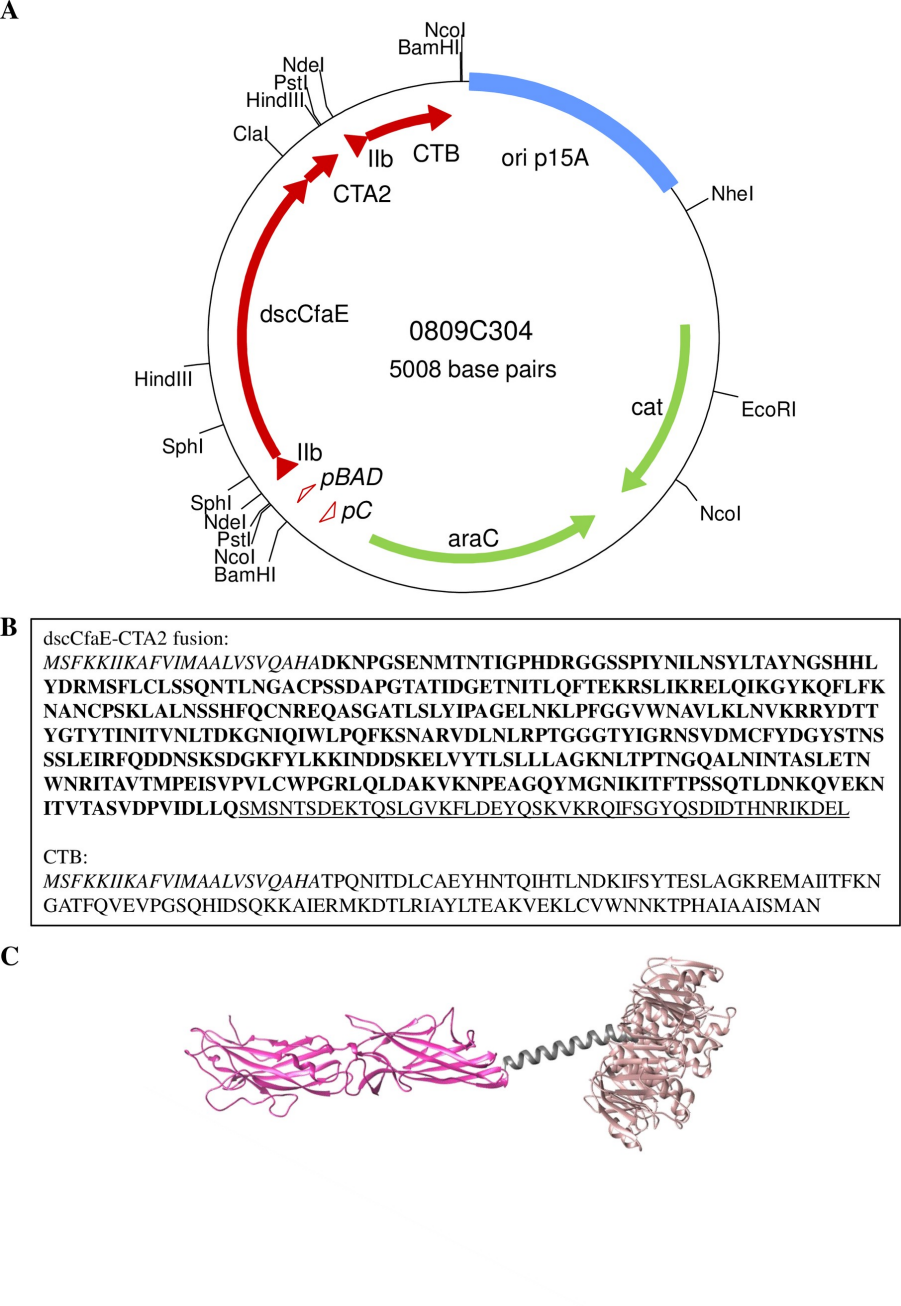
Construction of dscCfaE-CTA2/CTB chimera. (A) Map of 0809C304 plasmid. The dscCfaE, CTA2, and CTB genes are shown as red arrows, with the LTIIb-B gene signal peptide sequence shown as red triangles. The arabinose-inducible promoter (pBAD) and the araC promoter (pC) are shown as open red triangles Vector encoded araC (pBAD repressor) and cat (chloramphenicol resistance marker) are shown as green arrows. (B) Amino acid sequences for the dscCfaE-CTA2 and CTB genes. The LTIIb-B gene signal peptide sequence, which directs periplasmic export, is shown in italics, the amino acid sequence for mature dscCfaE is highlighted in bold, and the amino acid sequence for CTA2 is underlined. (C) Model of assembled dscCfaE-CTA2/CTB chimera. The dscCfaE-CTA2 fusion, which is noncovalently tethered to CTB (brown), is shown in pink (dscCfaE) and gray (CTA2). Model produced in UCSF Chimera software using crystal structures of dscCfaE (DOI: 10.2210/pdb2HB0/pdb) and cholera holotoxin (DOI: 10.2210/pdb1S5E/pdb).

The dscCfaE-CTA2/CTB expression plasmid, p0809C304, was made by insertion of the dscCfaE coding region within the multiple cloning site of pARLDR19 [36], which contain the CTA2 and CTB genes from *Vibrio cholerae* strain O395 [30, 37]. *Sph*I and *Cla*I sites were added to the 5’ and 3’ ends, respectively, of the coding region for mature dscCfaE by PCR amplification from pREP19 [13] using primers CFaEdscSphF and CFaEdscClaR. A schematic of the genes encoding dsc19CfaE-CTA2 and CTB, with restriction sites, is shown in Fig 1A. As the dscCfaE coding region has an internal *Sph*I site, a *Cla*I- partial *Sph*I digest of the PCR product was inserted into *Sph*I-*Cla*I cut pARLDR19. The mature dscCfaE portion of the CTA2 fusion begins with residue 2 and ends with DLLQ of the donor strand, followed by residues 194–240 of a C187S variant of CTA2 (SMSNTSD…KDEL) (Fig 1B). The CTB expression plasmid, p0826C1, was constructed from p0809C304 by self-ligation of the large *Nde*I fragment, which deletes coding sequences for the mature fusion protein and second LTIIb-B gene leader. Each plasmid, p0826C1 or p0809C304, was used to transform BL21 for protein expression.

### Protein expression and purification

**Recombinant CfaE.** His-tagged dscCfaE [also referred to as dsc_19_CfaE(His)_6_ or CfaE] protein was purified as previously described [13].

#### Recombinant dscCfaE-CTA2/CTB (chimera)

*E*. *coli* BL21 cells (Novagen, Madison, WI) containing p0809C304 were grown to late log phase at 25°C in APS^™^ superbroth (Difco, Detroit, MI) containing 25 μg/mL chloramphenicol. Protein expression was induced with 0.02% arabinose for three hours. Cells were harvested by centrifugation, resuspended 1:4 (w/v) with phosphate buffered saline (PBS) containing 5 mM imidazole, and lysed by two cycles of microfluidization (model M-110Y apparatus; Microfluidic Corp., Newton, Mass.). The lysate was centrifuged at 17000 x *g*. Supernatant was loaded onto a XK16 column (GE Healthcare, Piscataway, NJ) containing 10 mL of Talon® Superflow resin (Clontech Laboratories, Inc., Mountain View, CA). The column was washed with 10 mM imidazole and chimera was eluted with 50 mM imidazole. The eluate was pooled, diluted tenfold with 20 mM sodium phosphate buffer pH 6.5, and applied to a 5 ml HiTrap SP column (GE Healthcare). Chimera was eluted using a 0–400 mM sodium chloride (NaCl) linear gradient over 30 column volumes (CVs). Eluates were analyzed by sodium dodecyl sulfate (SDS)-polyacrylamide gel electrophoresis (PAGE) and western blot. Protein concentrations were determined by Pierce^™^ BCA protein assay Reagent (Thermo Fisher Scientific, Waltham, MA). Fractions containing chimera were pooled, sterilized by passage through a 0.22 μM filter (Millipore, Billerica, MA) and stored at -80°C.

#### Recombinant CTB

*E*. *coli* TE1 cells containing p0826C1 were grown as described for the chimera expression clone. Protein was purified from harvested cells using similar methods to the chimera, with the exception that CTB was eluted from the HiTrap SP column using a 0–500 mM NaCl linear gradient over 40 CVs.

### SDS-PAGE and Western blot analysis of dscCfaE-CTA2/CTB and component proteins

Proteins were denatured in SDS-PAGE loading buffer and heated at 100°C for 5 min. The denatured protein samples were separated by 15% SDS-PAGE. Gels were stained with GelCode^™^ Blue Stain Reagent (Thermo Fisher Scientific, Waltham, MA). For western blot analysis, proteins were electrotransferred to a nitrocellulose membrane after separation by SDS-PAGE. The presence of CfaE, CTA2 or CTB was detected by probing the membrane with polyclonal rabbit anti-serum raised against dscCfaE [13], CTA2, or CTB [36], followed by incubation with alkaline phosphatase-conjugated goat anti-rabbit antibodies (Caltag Laboratories, Burlingame, CA). Reactive protein bands were visualized using a NCIP/NBT colorimetric detection kit (Promega, Madison, WI).

### Detection of toxic activity in commercial CTB, recombinant CTB and chimera protein preparations

Native CTB (nCTB), purified from holotoxin-producing *V*. *cholerae* and specified to contain less than 0.5% holotoxin (determined by SDS-PAGE), was purchased from Sigma-Aldrich, St Louis, MO (Cat. No. C9903, Lot Nos. 044K4037 and 085K4153). The amount of active cholera holotoxin in commercial CTB, recombinant CTB, and the dscCfaE-CTA/CTB chimera was estimated by assaying serial 2-fold dilutions of known amounts of protein on mouse Y1 adrenal cells in 96 well format [40], which were compared to serial dilutions of purified cholera holotoxin. One toxic dose is the amount of protein required to cause 75–100% rounding of the cells, which corresponds to approximately 500 pg of holotoxin. For example, if 1 μg (10^6^ pg) of nCTB has 1 unit of toxicity, then we estimate it to contain the equivalent of 500/10^6^ or 0.05% active holotoxin.

### GM1 ganglioside binding assay

Nunc-immunosorp plates were coated with 100 μL per well with GM1 ganglioside (Sigma-Aldrich) diluted to 2 μg/ml in PBS. Plates were incubated at 37°C overnight. After washing, the plates were blocked with 200 μL/well of 1% bovine serum albumin (BSA, Sigma-Aldrich) in PBS at 37°C for 30 min. Plates were washed with PBS, and 100 μL of the following proteins (all diluted to 2 μg/ml in PBS) were added to duplicate wells: dscCfaE-CTA2/CTB, dscCfaE, and commercially available CTB (List Biologics). Plates were incubated for 1 hour and washed with PBS/Tween 20 (PBST). Polyclonal rabbit anti-CfaE antibody (100 μL/well of a 1:20,000 dilution) was added to the plates, which were incubated for 90 min and washed with PBST. Polyclonal goat anti-rabbit IgG-horseradish peroxidase (HRP) conjugate (Fitzgerald Industries Intl., Acton, MA) was diluted 1:5,000 in 0.1% BSA/PBST and added to the plates (100 μl/well). Plates were incubated for an additional 90 min and washed with PBST. Orthophenylenediamine substrate (Sigma-Aldrich), prepared as per manufacturer instructions, was added to the plates (100 μl/well). Plates were incubated for 20 min and optical densities at 450 nm (OD_450_) were measured. Results were adjusted for the blank and averaged. All incubations were performed at room temperature (RT). As a positive control for the assay, CTB bound to GM1 was confirmed by incubation with goat anti-CTB antibodies (List Biologics) as the primary antibody followed by detection with a rabbit anti-goat-HRP conjugate (KPL).

### Polystyrene bead adsorption and mannose-resistant hemagglutination (MRHA)

Purified protein preparations were adsorbed to 3 μm polystyrene beads (Polysciences, Inc.) using the manufacturer’s suggested protocol with modifications. Beads were washed 3 times with 1.5 mL 0.1M boric acid, pH 8.5 (borate buffer). Protein adsorption was performed in 0.2 mL volumes with the addition of 50 μg of dscCfaE-CTA2/CTB, dscCfaE, or CT (List Biologics). Adsorbed beads were blocked with borate buffer containing 0.05mg/ml of BSA. After blocking, beads were pelleted, resuspended in borate buffer, and tested for MRHA of bovine erythrocytes (Lampire Biological Laboratories, Pipersville, PA) using previously described methods using equal volumes (25 μL) of 3% erythrocytes, bead suspension, and phosphate buffered saline with 0.5% D-mannose [12].

### Immunization of mice and sample collection

Using methods similar to those previously described [15], female BALB/c mice, ages 6–8 weeks (Jackson Laboratory, Bar Harbor, ME) were quarantined for 1 week prior to study onset. Animals were housed in laminar flow cages and food and water were provided *ad libitum*. Mice in groups of 8–10 were immunized on days 0, 14 and 28 by the intranasal (IN) or orogastric (OG) routes with varying amounts of dscCfaE-CTA2/CTB (Table 1). Control animals were vaccinated with PBS, dscCfaE and/or CTB, which was purchased either commercially (IN studies; Cat. #C9903, Sigma-Aldrich) or purified as a recombinant protein (OG study). For IN immunizations, mice were anesthetized with Isoflurane (Halocarbon Products Corporation, River Edge NJ) and 20 μL of the immunizing preparation was administered to the external nares via pipette (10 μL per nare). For OG immunizations, gastric activity was neutralized with a 0.5 ml of 0.6 M sodium bicarbonate gavage. The immunizing preparation (in 0.5 ml) was then immediately administered (OG) through a sterile plastic feeding tube. Blood was collected prior to the first vaccination (day -3), and on days 13 and 27 via tail bleeding. On day 42, animals were anesthetized with Ketamine-Xylazine (Phoenix Scientific, Inc., St. Josephs MO), bled by cardiac puncture and then euthanized by cervical dislocation. Blood samples were allowed to clot and then centrifuged at 400 x g at 4°C for 15 min. Serum was then collected and stored at -20°C. Animal study protocol was reviewed and approved by the NMRC Institutional Animal Care and Use committee in compliance with all applicable Federal regulations governing the protection of animals in research.

**Table 1:** Murine experimental study designs.

Study^a^	Vaccine		Toxoid	
	Antigen (μg)	Dose (μg)	Antigen (μg)	Dose (μg)
**1**	dscCfaE-CTA2/CTB	65	-	-
	dscCfaE	25	CTB	36.5
	-	-	CTB	36.5
	dscCfaE	25	-	-
	- (PBS)	-	-	-
**2**	dscCfaE-CTA2/CTB	65	-	-
	dscCfaE-CTA2/CTB	21.3^b^	-	-
	dscCfaE-CTA2/CTB	7.1^b^	-	-
	dscCfaE-CTA2/CTB	2.4^b^	-	-
	-	-	CTB	13
	dscCfaE	25	-	-
	- (PBS)	-	-	-
**3**	dscCfaE-CTA2/CTB	162	-	-
	dscCfaE	62	CTB	91
	dscCfaE	62	-	-
	- (PBS)	-	-	-

^a^ Animals immunized by the IN route for Studies 1 and 2 and by the OG route for study 3.

^b^ For ease of reference in the manuscript, these doses were rounded to the nearest μg to 21, 7 and 2 respectively.

### Serum antibody titer determination in mouse serum samples

Serum IgG and IgA antibodies to dscCfaE were detected by enzyme-linked immunosorbent assays (ELISA) as previously described [15]. Detection of IgG and IgA antibodies to CTB proceeded with the same protocol, with the following exceptions. Nunc flat-bottom 96-well plates were initially coated with 0.5 μg GM1 ganglioside/ml for 60 min at 37°C then overnight at 4°C, and blocked with 1% Fetal Calf Serum (Serologicals, Atlanta, GA)/PBS (Diamedix, Miami, FL)/0.05% Tween 20 (FCS-PBST, Sigma-Aldrich). Plates were then coated with 100 μL of 0.5 μg/ml CTB diluted in PBS, and incubated for 60 min at 37°C. Serum samples and antibody reagents were diluted in 1% FCS-PBST and 0.1% FCS-PBST, respectively. Plates were incubated with HRP-conjugated goat anti-mouse IgG and IgA antibodies for 90 min at RT, developed with 2,2'-azino-bis(3-ethylbenzthiazoline-6-sulphonic acid) (Kirkegaard & Perry Laboratories, Milford, MA), and read at A_405_. Reciprocal titers were calculated as previously described [12]. For both anti-CfaE and anti-CTB ELISA data, reciprocal titers <50, the lowest dilution tested, were assigned values of 25 for computational purposes and final titers represent the mean of duplicate analyses performed on separate days.

### Antibody-mediated hemagglutination inhibition assay

A hemagglutination inhibition (HAI) assay was used as the proxy for neutralization of fimbrial adhesin-mediated adhesion. Serum samples were tested as previously described [12]. CFA/I^+^ ETEC strain H10407 and bovine erythrocytes (Lampire Biological Laboratories, Pipersville, PA) were used in these assays. HAI titer was defined as the reciprocal of the highest dilution of serum that inhibited agglutination. Values below 1:32, the lowest dilution tested, were assigned a reciprocal titer of 16 for computational purposes.

### Statistical and graphical analyses

Mouse HAI and ELISA titers were log_10_-transformed for statistical analysis and group titers were compared using a one-way analysis of variance (ANOVA) and a Tukey’s post hoc test was used for pair-wise comparisons. All statistical analyses were performed using GraphPad Prism Version 6.07 for Windows (Graphpad Software, San Diego, CA), and a *P* value of <0.05 was considered significant.

## Results

### Purification and characterization of dscCfaE-CTA2/CTB chimera

**Fig 2:**
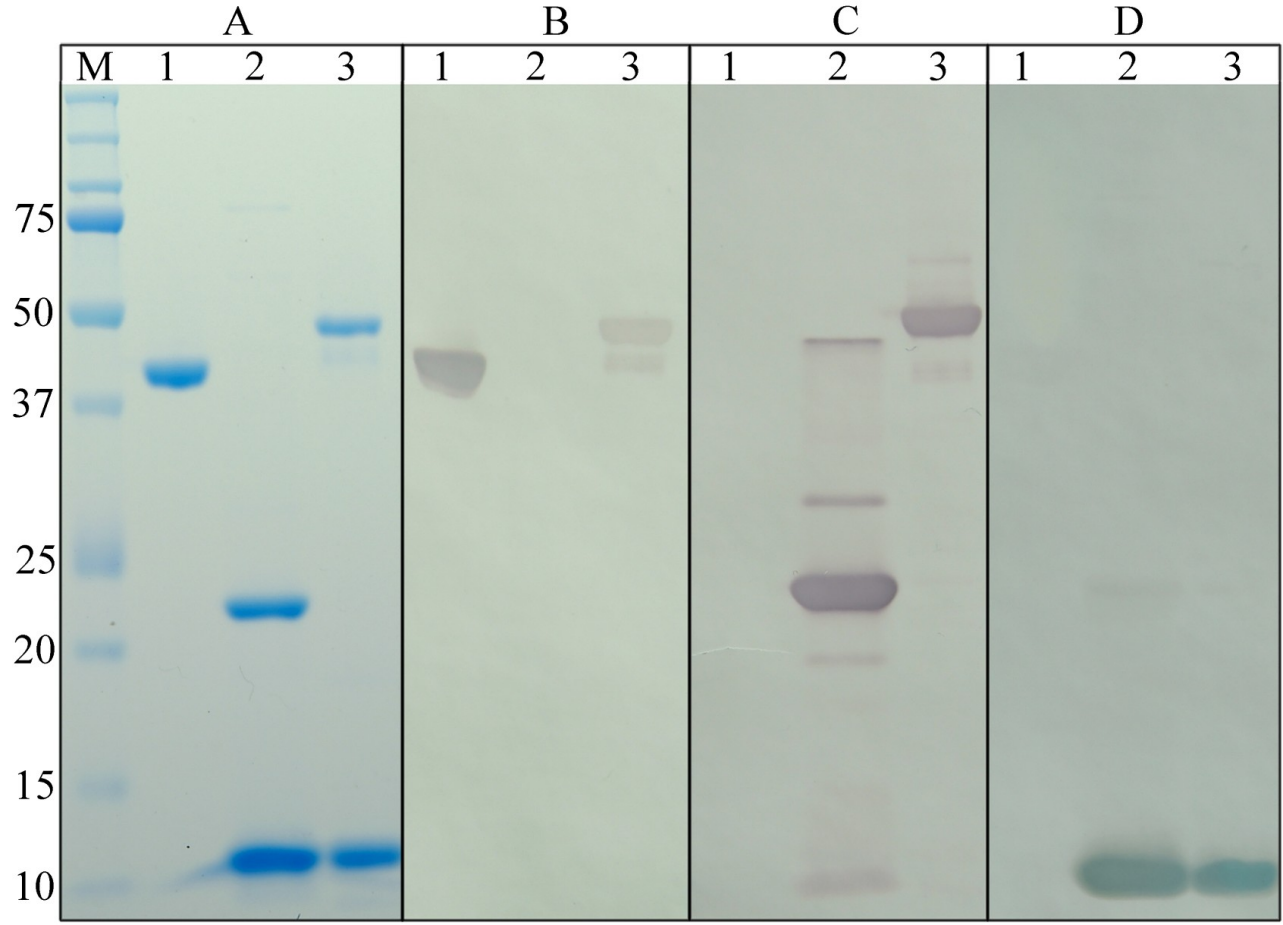
Characterization of the protein components of the dscCfaE-CTA2/CTB chimera. Recombinant dscCfaE (Lane 1), CT (Lane 2) and dscCfaE-CTA2/CTB (Lane 3) separated by SDS-PAGE (A) and immunodetected with anti-CfaE (B), anti-CTA (C), or anti-CTB polyclonal rabbit serum (D). The molecular weight marker (Bio-Rad Precision Plus Protein^™^ Standards) is shown in Lane M.

The vector p0809C304 (Fig 1A), coding for dscCfaE-CTA2/CTB (chimera), was transformed into BL21 *E*. *coli* cells for expression. Chimera was purified to >90% purity from harvested bacterial cell paste using a two-step column chromatography process. SDS-PAGE of the purified chimera identified two primary protein bands with molecular weights of 45 kDa and 12 kDa, corresponding to the approximate sizes of the dscCfaE-CTA2 fusion and CTB monomer respectively. A faint protein band with a molecular weight of 42 kDa was also observed. (Fig 2A, Lane 3). Identity of the protein bands was confirmed through Western blotting. As expected, the dscCfaE-CTA2 fusion component of the chimera reacted with anti-CfaE antibodies, as well as with antibodies generated to CTA, while the CTB component reacted solely with anti-CTB antibodies (Fig 2B–2D, Lane 3). The 42 kDa minor protein band reacted with both anti-CfaE and anti-CTA antibodies, suggesting that the band is a proteolytic product of the dscCfaE-CTA2 fusion (Fig 2B–2D, Lane 3).

### Analysis of *in vitro* GM1 ganglioside binding properties of the dscCfaE-CTA2/CTB chimera

**Fig 3:**
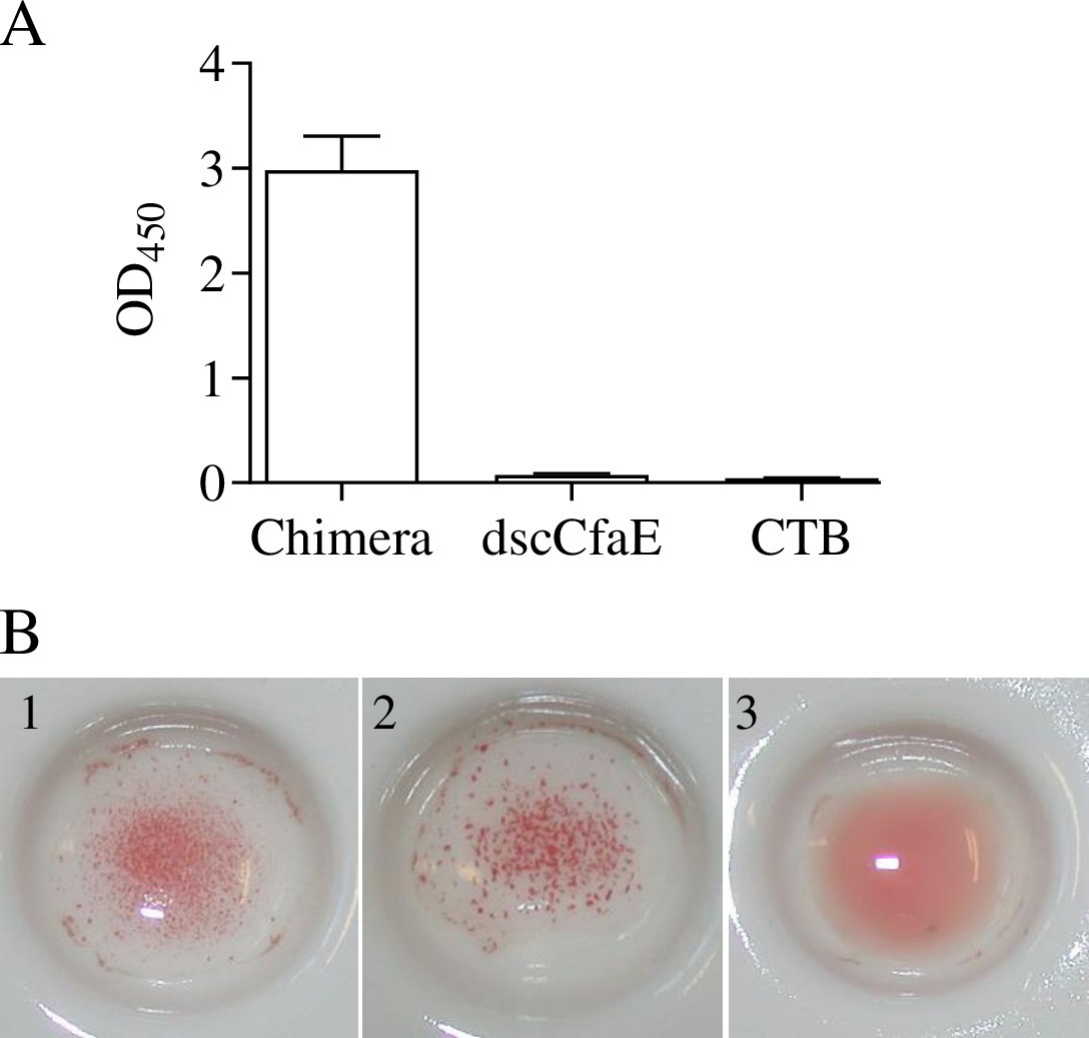
Binding functionalities of the dscCfaE-CTA2/CTB chimera. (A) Binding of chimera protein to solid-phase GM1 by ELISA. GM1-coated plates were incubated with chimera, dscCfaE, or CTB. Rabbit anti-dscCfaE and goat anti-rabbit IgG-HRP antibodies were used as the primary and secondary antibody, respectively. The bars represent mean absorbance at OD 450nm (+SEM of 4 replicate wells from one representative experiment). (B) *In vitro* adherence of chimera to bovine erythrocytes, measured by visual hemagglutination. Polystyrene beads were coated with chimera (1), dscCfaE (2) or CTB (3) and incubated with bovine erythrocytes.

Chimera assembly and GM1 ganglioside binding functionality, via the CTB pentamer, was determined using an *in vitro* ELISA-based GM1 ganglioside binding assay. As shown in Fig 3A, the chimera bound to GM1, resulting in a strong OD_450_ signal when detected by anti-CfaE antibody. When either CTB or dscCfaE alone was incubated with the GM-1 coated wells, the OD_450_ signal was significantly lower than observed with the chimera (*P*<0.001). CTB binding to GM1 would not be detected with the anti-CfaE antibody. In combination, these data suggest that the two components of the chimera, dscCfaE and CTB, are assembled and that its CTB component was able to mediate the binding of the chimera to GM1 ganglioside.

### Analysis of *in vitro* adhesive properties of dscCfaE-CTA2/CTB chimera

As reported previously, dscCfaE is able to mediate the mannose-resistant agglutination (MHRA) of bovine erythrocytes when absorbed to polystyrene beads [13]. To determine whether the functionality of dscCfaE is retained in the context of the chimera, dscCfaE, CTB, and the chimera were adsorbed onto polystyrene beads, which were then incubated with bovine erythrocytes. As shown in Fig 3B, both the chimera and dscCfaE were positive for MRHA, while CT was MRHA negative, confirming that, in the chimera, dscCfaE retains its functional binding activity.

### Toxicity of commercial CTB, recombinant CTB and dscCfaE-CTA2/CTB chimera

To confirm that the chimera did not have any toxic activity, purified proteins were assayed in the Y1 adrenal cell assay. Neither dscCfaE-CTA2/CTB nor recombinant CTB showed any detectable rounding of the cells at the maximum amount of protein added (3.7 and 4.8 μg respectively) and thus had no detectable toxic activity, while 500 pg of the positive control cholera holotoxin caused 75–100% rounding of the cells. Two commercial batches of CTB (Sigma, C9903, lot numbers 044K4037 and 085K4153) that were used in admixture controls were tested and caused full rounding of the cells at 195 and 100 ng per well respectively, estimated to be equivalent to 0.26 and 0.5% holotoxin contamination, respectively.

### Intranasal immunization of mice with the chimera, dscCfaE, and CTB

**Fig 4:**
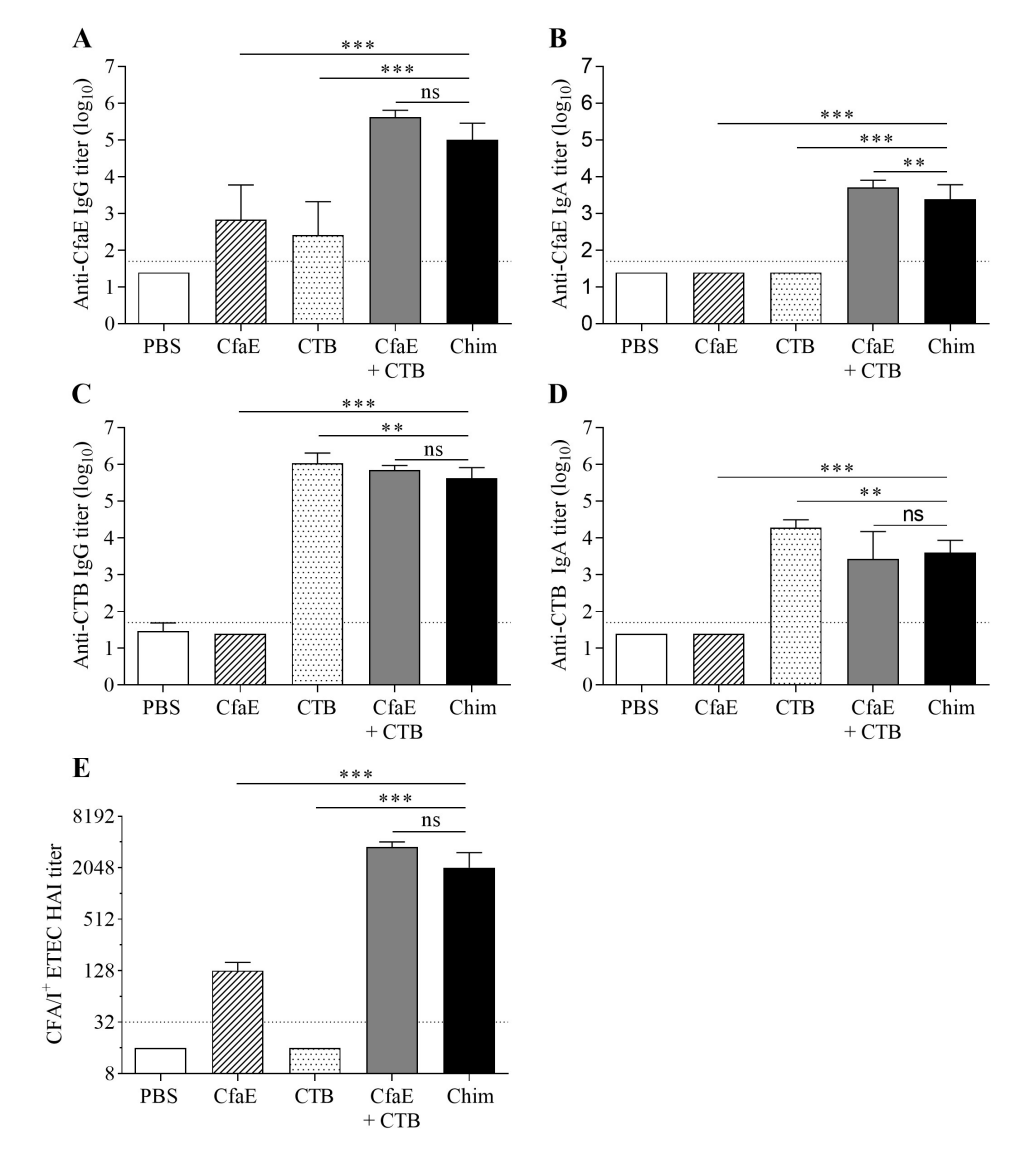
Intranasal immunization with dscCfaE-CTA2/CTB chimera (Chim). Serum antibody responses on day 42 in BALB/c mice vaccinated IN with 65 μg dscCfaE-CTA2/CTB (black bars), 25 μg CfaE + 36.5 μg CTB (grey bars), 36.5 μg CTB (stippled bars), 25 μg CfaE (striped bars), or PBS (white bars). (A) Anti-CfaE IgG, (B) anti-CfaE IgA, (C) anti-CTB IgG and (D) anti-CTB IgA end point ELISA titers are shown as mean log_10_ titers ± SD. The horizontal lines (1.7) denote the lowest dilution tested. (E) Functional antibody titers are shown, as measured by the HAI assay using CFA/I-ETEC (H10407) and red blood cells. Data are represented as the median and interquartile ranges of data from each group and the horizontal dotted line (1:32) denotes the lowest dilution of serum tested. For A-E, statistical significance was determined by using one-way ANOVA and Tukey’s multiple comparisons test. Asterisks denote a significant difference between responses in the indicated groups (*P<0.05; **P<0.01; ***P<0.001; not significant (ns) *P*>0.05).

Groups of 8–10 mice were immunized intranasally with 65 μg of chimera, 25 μg of dscCfaE (CfaE), 36.5 μg CTB, an admixture containing 25 μg of CfaE and 36.5 μg CTB, or PBS. The amounts of antigen in the groups receiving CfaE or CTB alone were molar matched to the dose of the chimera and reflected the approximate amount of each antigen present in 65 μg of chimera. Immunization with chimera resulted in maximal anti-CfaE titers by day 42, two weeks after the third vaccination and analyses were performed on samples from this time point. Serum anti-adhesin IgG antibody titers were observed in both the chimera group and control groups, with titer levels significantly greater than those observed in the PBS group (all *P*<0.01) (Fig 4A and S1 Table). While anti-CfaE IgG titers were significantly greater in the chimera group compared to the CfaE alone and CTB alone control groups (both *P*<0.001), there was no significant difference between the chimera group and the group given an admixture of CfaE and CTB. Only the chimera and admixture groups exhibited anti-CfaE IgA titers significantly greater than the PBS control (*P*<0.001), and those titers in the chimera group were significantly lower than those in the admixture control (*P*<0.01) (Fig 4B).

Groups immunized with chimera, CTB, or an admixture of CfaE and CTB demonstrated strong serum antibody responses against CTB (Fig 4C and 4D) and both IgG and IgA anti-CTB titers in these groups were significantly greater than those of the PBS groups (all *P*<0.001). There was no significant difference in anti-CTB IgG and IgA titers between the chimera and admixture groups. However, levels in the CTB alone group were significantly greater than those in the chimera group for both assays (*P*<0.01).

Groups receiving CfaE, either alone, admixed with CTB or incorporated in the chimera, exhibited HAI titers significantly greater than the PBS control (Fig 4E, *P*<0.001). Titers were greater in the chimera group compared to the CfaE alone group (*P*<0.001), but not different from the admixture group. The lack of a detectable HAI activity in the serum from the group immunized with CTB alone suggests that the low anti-CfaE IgG titer detected in the serum by ELISA (Fig 4A) was likely an assay artifact.

### Immune response elicited by intranasal vaccination of mice with the chimera is dose-dependent

**Fig 5:**
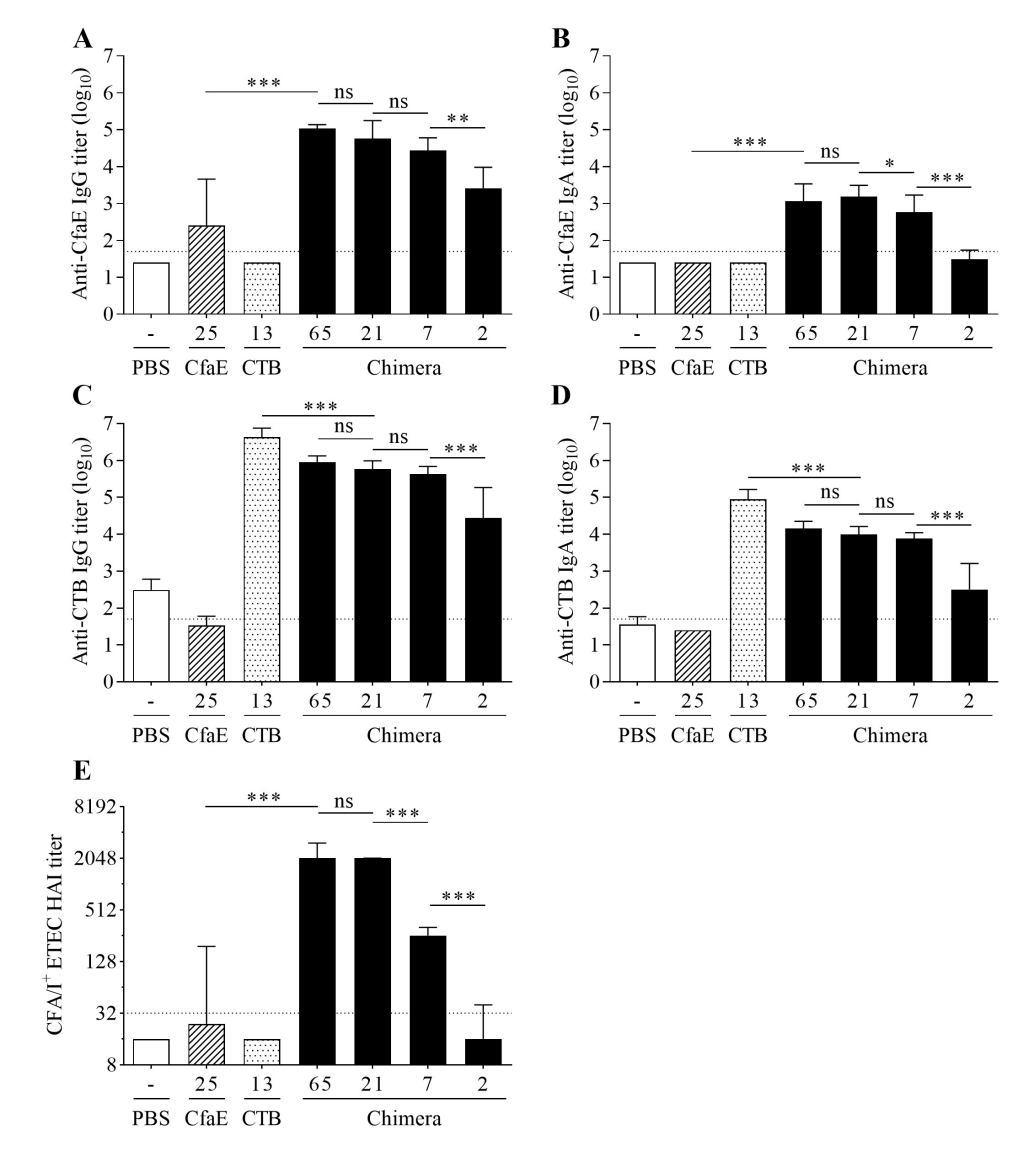
Dose-dependent immune response elicited by the dscCfaE-CTA2/CTB chimera. Serum antibody responses on day 42 in BALB/c mice vaccinated IN with 65 μg, 21 μg, 7 μg, or 2 μg, dscCfaE-CTA2/CTB (black bars), 13 μg CTB (stippled bars), 25 μg CfaE (striped bars), or PBS (white bars). (A) Anti-CfaE IgG, (B) anti-CfaE IgA, (C) anti-CTB IgG and (D) anti-CTB IgA end point ELISA titers are shown as mean log_10_ titers ± SD. The horizontal lines (1.7) denote the lowest dilution tested. (E) Functional antibody titers are shown, as measured by the HAI assay using CFA/I-ETEC (H10407) and red blood cells. Data are represented as the median and interquartile ranges of data from each group and the horizontal dotted line (1:32) denotes the lowest dilution of serum tested. For A-E, statistical significance was determined by using one-way ANOVA and Tukey’s multiple comparisons test. Asterisks denote a significant difference between responses in the indicated groups (*P<0.05; **P<0.01; ***P<0.001; ns—not significant).

Mice immunized IN with decreasing amounts of chimera exhibited clear dose-dependent responses with respect to anti-CfaE, anti-CTB and HAI titers (Fig 5 and S1 Table). All four doses of chimera elicited anti-CfaE IgG titers that were significantly greater than those of the PBS control group (*P*<0.001) (Fig 5A). Each successively lower dose elicited a lower titer, though only the lowest two doses elicited titers significantly different from each other (*P*<0.01). A similar pattern of responses was observed for anti-CfaE IgA titers, with significant differences in titers between the 21 μg and 7 μg dose groups (*P*<0.05) and the 7 μg and 2 μg dose groups (*P*<0.001). Only the three highest chimera doses elicited anti-CfaE IgA titers significantly greater than those of the PBS group (*P*<0.001). For both IgG and IgA responses, titers in the 65 μg dose chimera group were significantly greater than those receiving dscCfaE (CfaE) alone, though both received approximately the same quantity of adhesin (25 μg) (both *P*<0.001). Groups immunized with varying doses of chimera or CTB alone exhibited strong serum antibody responses against CTB (Fig 5C and 5D), with both the IgG and IgA titers being significantly greater than those of the PBS groups (all *P*<0.001). There was a clear dose-dependent trend in titers among the four chimera dose groups, though only the lowest two dose groups had anti-CTB titers IgG and IgA significantly different from each other (*P*<0.001). For both IgG and IgA anti-CTB responses, titers in the 21 μg dose chimera group were significantly less than those in the CTB alone group, though both received approximately the same quantity of toxoid (13 μg) (both *P*<0.001).

Serum HAI titers for the groups receiving the upper three doses of chimera (65, 21 and 7 μg) were significantly higher than those in the PBS control group (Fig 5E) (all *P*<0.001). Decreasing the dose of chimera resulted in lower HAI titers, with significant differences in titers between the 21 and 7 μg dose groups (*P*<0.001), as well as the 7 μg and 2 μg dose groups (*P*<0.001). Finally, mice receiving 65 μg of chimera expressed significantly higher HAI titers than those receiving CfaE alone (*P*<0.001), though both groups received the same amount of adhesin (25 μg).

### Immunization with chimera by the orogastric route

**Fig 6:**
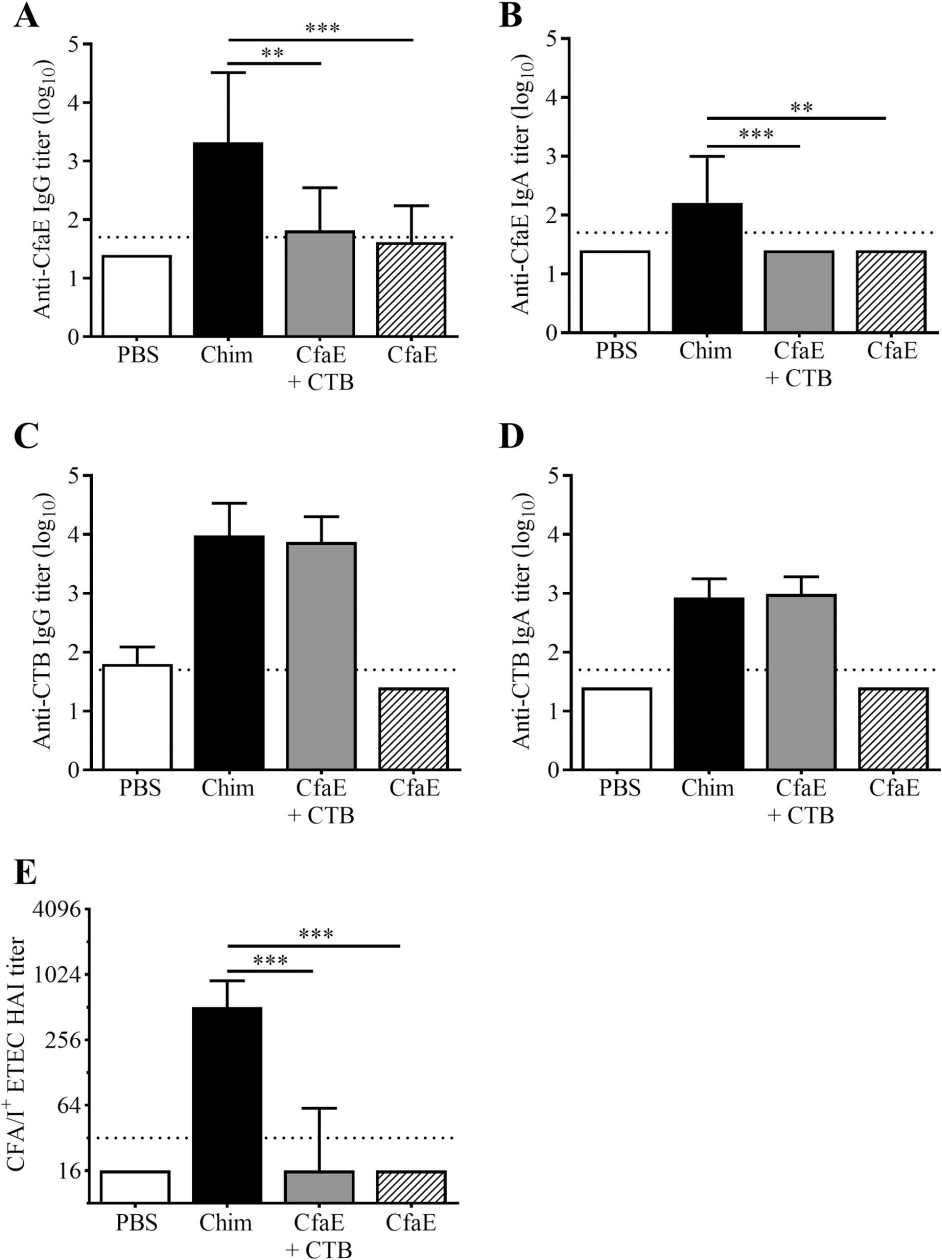
Orogastric immunization with dscCfaE-CTA2/CTB chimera (Chim). Serum antibody responses on day 42 in BALB/c mice vaccinated orogastrically with 162 μg dscCfaE-CTA2/CTB (black bars), CfaE + CTB admixture containing 62 μg dscCfaE and 91 μg CTB (grey bars), 62 μg CfaE alone (striped bars), or PBS (white bar). The admixture and CfaE groups were molar matched to the approximate amounts present in 162 μg chimera. (A) Anti-CfaE IgG, (B) anti-CfaE IgA, (C) anti-CTB IgG and (D) anti-CTB IgA end point ELISA titers are shown as mean log_10_ titers ± SD. The horizontal lines (1.7) denote the lowest dilution tested. (E) Functional antibody titers are shown, as measured by the HAI assay using CFA/I-ETEC (H10407) and red blood cells. Data are represented as the median and interquartile ranges of data from each group and the horizontal dotted line (1:32) denotes the lowest dilution of serum tested. For A-E, statistical significance was determined by using one-way ANOVA and Tukey’s multiple comparisons test. Asterisks denote a significant difference between responses in the indicated groups (**P<0.01; ***P<0.001).

Oral immunization with the dscCfaE-CTA2/CTB chimera led to higher anti-CfaE responses in comparison to dscCfaE (CfaE) + CTB or CfaE alone (Fig 6). The chimera elicited significantly higher anti-CfaE IgG and IgA titers in comparison to CfaE + CTB or CfaE alone (*P*<0.01 for all comparisons; Fig 6A and 6B). Anti-CTB IgG and IgA responses are similar among all groups that received chimera or CfaE + CTB (Fig 6C and 6D). The functional responses, as measured by HAI titer, mirrored the anti-CfaE IgG titers and mice that received the chimera had significantly higher HAI titers in comparison to CfaE + CTB or CfaE alone (*P*<0.001; Fig 6E).

## Discussion

In this study, we investigated the potential for boosting the immunogenicity of dscCfaE without the use of an exogenous adjuvant. To achieve this, we constructed and purified a CT-like chimeric protein, dscCfaE-CTA2/CTB, in which the CTA1 subunit of CT was substituted with dscCfaE, a strategy described previously to increase the immunogenicity of other soluble antigens [32–36]. We demonstrated that the dscCfaE-CTA2 and CTB components assembled into a non-covalent complex that retains the GM1-ganglioside binding activity of the CTB pentamer and the ability to induce the MRHA of bovine erythrocytes when adsorbed to polystyrene beads, a phenotype first attributed to CFA/I by Evans *et al*. and later, to the CFA/I tip-adhesin, CfaE [13, 41, 42]. The intact binding functionalities of CTB and dscCfaE indicated that the two proteins were in their native, active conformations in the chimera construct and that conformational epitopes were preserved. The immunogenicity of the chimera protein, when given IN, was evaluated in a BALB/c mouse model. In this model, we observed that a dose as low as 2 μg of chimera, which contained the equivalent of approximately 1 μg of dscCfaE, elicited a significantly greater serum anti-dscCfaE IgG immune response than immunization with 25 μg of dscCfaE alone. This demonstrated that the delivery of the dscCfaE antigen with the mucosal adjuvant, CTB, in the context of the chimera, increased the response to small amounts of dscCfaE without the need of an exogenous adjuvant. In addition, we observed that immunization with chimera generated a robust serum immune response to CTB. As CTB is immunologically cross-reactive to LTB, the B subunit from LT, an immune response generated to CTB would potentially contribute to protection against diarrhea caused by LT-producing ETEC, as seen previously with a CTB-containing whole cell cholera vaccine [29, 43]. We also demonstrated that the immune response against CfaE generated by immunizing with the chimera was neutralizing, as determined by measuring the inhibition of CfaE-mediated hemagglutination of CFA/I^+^ H10407 ETEC by serum from immunized mice. The results clearly show that the incorporation of the CfaE protein into the chimera has not impaired its immunogenic characteristics and that the neutralizing epitopes of CfaE remain in the proper conformation.

We also evaluated the immunogenicity of an admixture of dscCfaE and CTB, which contained molar equivalent amounts of the two antigens found in 65 μg of the chimera. Comparable to the chimera, the IN immunization of mice with the admixture elicited a robust, anti-adhesin neutralizing response. The strong immunogenicity of the admixture was not completely unexpected, as the ability of CTB to adjuvant immune responses to non-coupled antigens when administered IN has been well documented [24, 44]. However, we had hoped to observe a stronger response elicited by the chimera. A likely explanation for the observed high responses elicited by the admixture was that low levels of intact CT (~0.27–0.5% holotoxin) were later detected in the commercially-purchased CTB used to prepare the admixture formulations in the two IN studies. As intact CT is a potent adjuvant [25], the low level of contaminating holotoxin could have artificially boosted responses observed in the admixture group, masking any potential advantage that the chimera formulation may have demonstrated.

The IN route was used in the initial murine studies to provide an initial screen of the chimera, as we have previously observed strong immune responses to ETEC antigens elicited by this route [15]. However, due to the risk of Bell’s palsy associated with IN delivery of vaccine antigens [27, 45], we wanted to evaluate other, more clinically acceptable routes of administration. It has been reported that co-administered CTB is a poor adjuvant by the oral route [24, 46]. We also observed in a previous *A*. *nancymaae* non-human primate study that co-administered CTB, which enhanced the immunogenicity of dscCfaE given IN, failed to adjuvant the serum anti-CfaE response by the OG route [15]. Thus, although the dscCfaE + CTB admixture may have performed comparably to the chimera by the IN route, the chimera, which physically links the two molecules, may be a superior formulation when administered by the other routes by facilitating the presentation of the attached antigen to the immune system [47]. We explored this by immunizing mice with the dscCfaE-CTA2/CTB chimera via the OG route and found that, by this route, the chimera elicited higher anti-CfaE titers than an admixture containing similar amounts of CfaE and recombinant CTB (no contaminating holotoxin). Additionally, the higher anti-CfaE titers corresponded to significantly higher HAI titers in the mice receiving the chimera. Thus, by the OG route, the chimera was superior to the admixture in enhancing the immune response to the CfaE antigen. However, the anti-CfaE IgG and HAI titers were lower overall compared to those observed with IN immunization.

In summary, we have shown that the incorporation of an ETEC fimbrial adhesin into a CT-like chimera is an effective strategy for improving the immunogenicity of CfaE by directing antigen uptake through the GM1 binding activity of CTB, eliminating the requirement for an exogenous adjuvant. Furthermore, as well as functioning as an adjuvant, the CTB unit of the chimera could also induce a cross-reactive response against the LT-toxin produced by some ETEC strains. This response could potentially act in synergy with the anti-adhesin response to protect against disease caused by LT-expressing ETEC. The combination of an ETEC fimbrial adhesin and an LT-related antigen into one molecular complex eliminates the need for an additional LT-like vaccine component, reducing manufacturing costs and valency. The findings presented herein show that ETEC adhesin-CTB chimeras are immunogenic and viable candidates for inclusion in a multivalent ETEC vaccine strategy, which would consist of chimeras containing epidemiologically prevalent ETEC CF subunits to boost vaccine coverage. However, the efficacy of the immune responses they elicit needs to be assessed. Unfortunately, a robust murine model for assessing ETEC-mediated diarrhea does not currently exist. Future efforts will focus on determining the efficacy of immunization with the CFA/I derived dscCfaE-CTA2/CTB adhesin chimera in a previously developed *Aotus nancymaee* non-human primate challenge model [17]. Using this model, we will assess whether immunization with the chimera will protect against diarrhea following challenge with the CFA/I-expressing ETEC strain H10407.

## Supporting information

S1 Table Serum antibody and HAI titers in mice vaccinated with dscCfaE-CTA2/CTB and its components. (DOCX)

S1 Fig Raw image of recombinant dscCfaE (Lane 1), CT (Lane 2) and dscCfaE-CTA2/CTB (Lane 3) separated by SDS-PAGE. The molecular weight marker (Bio-Rad Precision Plus Protein^™^ Standards), with the molecular masses of the individual standards, is shown in Lane M.
(PPTX)

S2 Fig Raw image of recombinant dscCfaE (Lane 1), CT (Lane 2) and dscCfaE-CTA2/CTB (Lane 3) separated by SDS-PAGE and immunodetected with anti-CfaE polyclonal rabbit serum. The molecular weight marker (Bio-Rad Precision Plus Protein^™^ Standards), with the molecular masses of the individual standards, is shown in Lane M.
(PPTX)

S3 Fig Raw image of recombinant dscCfaE (Lane 1), CT (Lane 2) and dscCfaE-CTA2/CTB (Lane 3) separated by SDS-PAGE and immunodetected with anti-CTA polyclonal rabbit serum. The molecular weight marker (Bio-Rad Precision Plus Protein^™^ Standards), with the molecular masses of the individual standards, is shown in Lane M.
(PPTX)

S4 Fig Raw image of recombinant dscCfaE (Lane 1), CT (Lane 2) and dscCfaE-CTA2/CTB (Lane 3) separated by SDS-PAGE and immunodetected with anti-CTB polyclonal rabbit serum. The molecular weight marker (Bio-Rad Precision Plus Protein^™^ Standards), with the molecular masses of the individual standards, is shown in Lane M.
(PPTX)

S1 File (PDF)
