# Supplementary material for: Biochemical and immunological characterization of an ETEC CFA/I adhesin cholera toxin B subunit chimera
Source: PLoS One. 2020 Mar 16;15(3):e0230138. doi: 10.1371/journal.pone.0230138 (PMC7075575; doi:10.1371/journal.pone.0230138)
Supplement: S1 Table — (DOCX) [file pone.0230138.s001.docx]

**S1 Table. Serum antibody and HAI titers in mice vaccinated with dscCfaE-CTA2/CTB and its components.**

| Study^a^ | Vaccine | | Toxoid | | Mean log_10_ Serum Antibody Titer (SD) | | | | Median HAI Titers (IQR) |
| --- | --- | --- | --- | --- | --- | --- | --- | --- | --- |
|  | Antigen (µg) | Dose (µg) | Antigen (µg) | Dose (µg) | α-CfaE IgG | α-CfaE IgA | α-CTB IgG | α-CTB IgA |  |
| 1 | dscCfaE-CTA2/CTB | 65 | - | - | 5.000 (0.4570) | 3.391 (0.3895) | 5.629 (0.2839) | 3.600 (0.3339) | 2048  (1920-3072) |
|  | dscCfaE | 25 | CTB | 36.5 | 5.621 (0.1869) | 3.707 (0.1967) | 5.847 (0.1239) | 3.425 (0.7508) | 3584  (2048-4096) |
|  | - | - | CTB | 36.5 | 2.414 (0.9075) | 1.4 (0) | 6.032 (0.2778) | 4.281 (0.2119) | 16 (0) |
|  | dscCfaE | 25 | - | - | 2.832 (0.9445) | 1.4 (0) | 1.4 (0) | 1.4 (0) | 128  (40-160) |
|  | - | - | - | - | 1.4 (0) | 1.4 (0) | 1.470 (0.2214) | 1.4 (0) | 16 (0) |
| 2 | dscCfaE-CTA2/CTB | 65 | - | - | 5.031 (0.1035) | 3.068 (0.4670) | 5.949 (0.1741) | 4.157 (0.1907) | 2048  (2048-3072) |
|  | dscCfaE-CTA2/CTB | 21.3^b^ | - | - | 4.762 (0.4846) | 3.188 (0.3050) | 5.760 (0.2284) | 3.992 (0.2163) | 2048  (1024-2048) |
|  | dscCfaE-CTA2/CTB | 7.1^b^ | - | - | 4.436 (0.3463) | 2.766 (0.4619) | 5.635 (0.2030) | 3.881 (0.1618) | 256  (128-320) |
|  | dscCfaE-CTA2/CTB | 2.4^b^ | - | - | 3.415 (0.5691) | 1.484 (0.2533) | 4.444 (0.8212) | 2.498 (0.7086) | 16  (16-40) |
|  | - | - | CTB | 13 | 1.4 (0) | 1.4 (0) | 6.635 (0.2399) | 4.948 (0.2632) | 16 (0) |
|  | dscCfaE | 25 | - | - | 2.404 (1.254) | 1.4 (0) | 1.526 (0.2504) | 1.4 (0) | 24  (16-192) |
|  | - | - | - | - | 1.4 (0) | 1.4 (0) | 2.489 (0.2916) | 1.556 (0.2074) | 16 (0) |
| 3 | dscCfaE-CTA2/CTB | 162 | - |  | 3.323 (1.193) | 2.212 (0.7857) | 3.981 (0.5485) | 2.933 (0.3137) | 512  (192-896) |
|  | dscCfaE | 62 | CTB | 91 | 1.820 (0.7288) | 1.4 (0) | 3.874 (0.4274) | 2.990 (0.2904) | 16  (16-60) |
|  | dscCfaE | 62 | - | - | 1.619 (0.6205) | 1.4 (0) | 1.4 (0) | 1.4 (0) | 16 (0) |
|  | - | - | - | - | 1.4 (0) | 1.4 (0) | 1.802 (0.2885) | 1.4 (0) | 16 (0) |

^a^ Animals immunized by the IN route for Studies 1 and 2 and by the OG route for study 3.

^b^ For ease of reference in the manuscript, these doses were rounded to the nearest µg to 21, 7 and 2 respectively.
