# Supplementary figures and images for: Biochemical and immunological characterization of an ETEC CFA/I adhesin cholera toxin B subunit chimera
Source: PLoS One. 2020 Mar 16;15(3):e0230138. doi: 10.1371/journal.pone.0230138 (PMC7075575; doi:10.1371/journal.pone.0230138)

## Slide 1
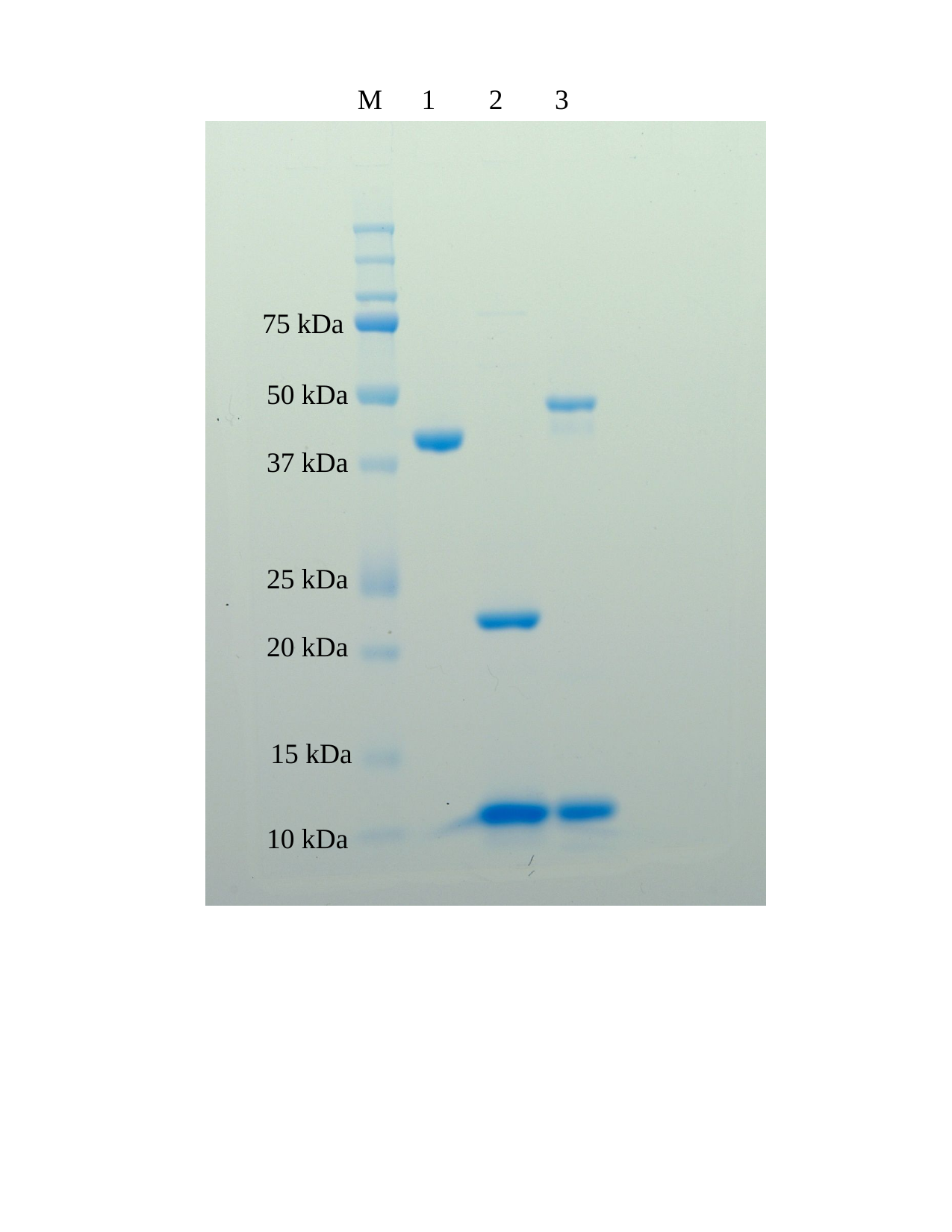

M
1
2
3
75 kDa
50 kDa
37 kDa
25 kDa
20 kDa
15 kDa
10 kDa

Supplement: S1 Fig — The molecular weight marker (Bio-Rad Precision Plus Protein™ Standards), with the molecular masses of the individual standards, is shown in Lane M. (PPTX) [file pone.0230138.s002.pptx]

## Slide 1
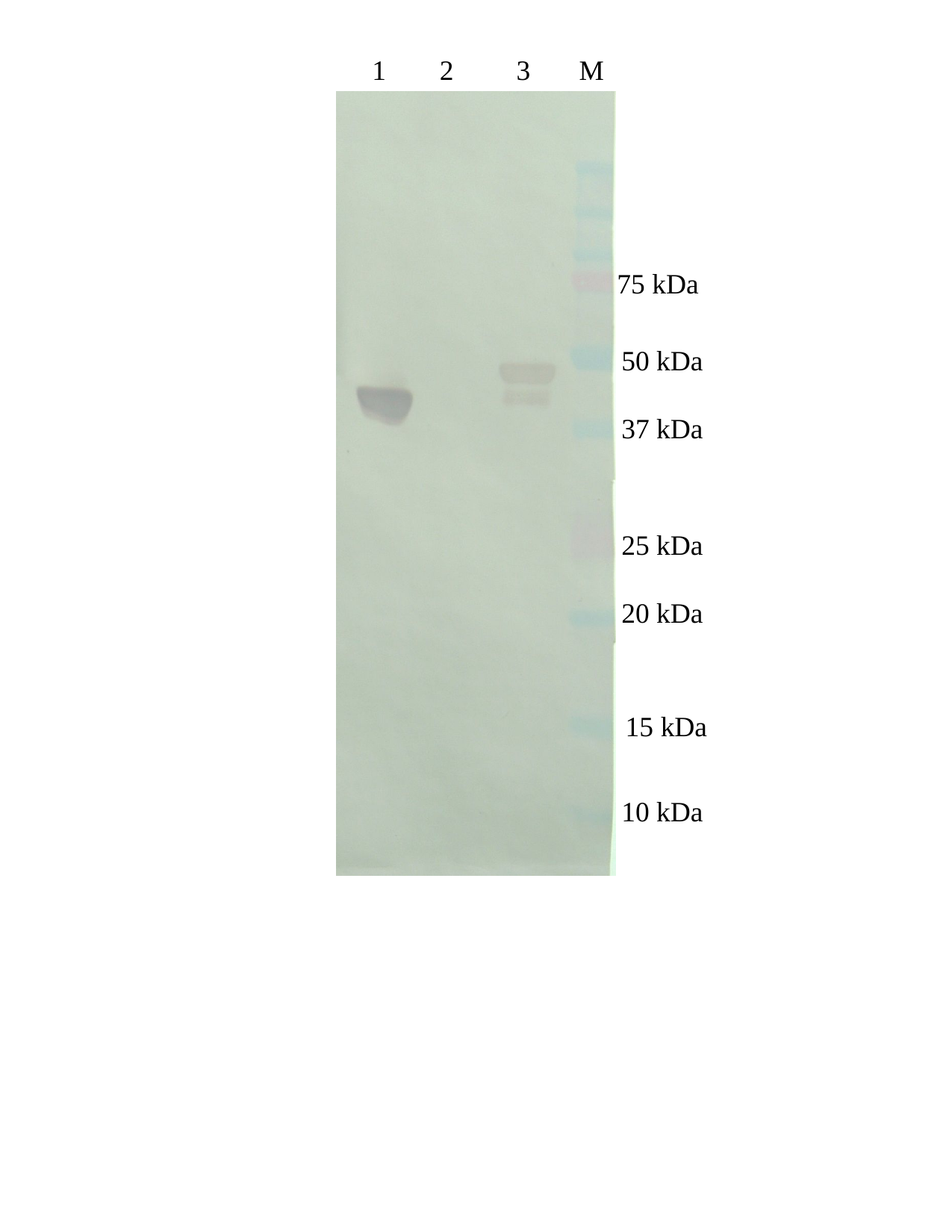

1
 2
3
M
75 kDa
50 kDa
37 kDa
25 kDa
20 kDa
15 kDa
10 kDa

Supplement: S2 Fig — The molecular weight marker (Bio-Rad Precision Plus Protein™ Standards), with the molecular masses of the individual standards, is shown in Lane M. (PPTX) [file pone.0230138.s003.pptx]

## Slide 1
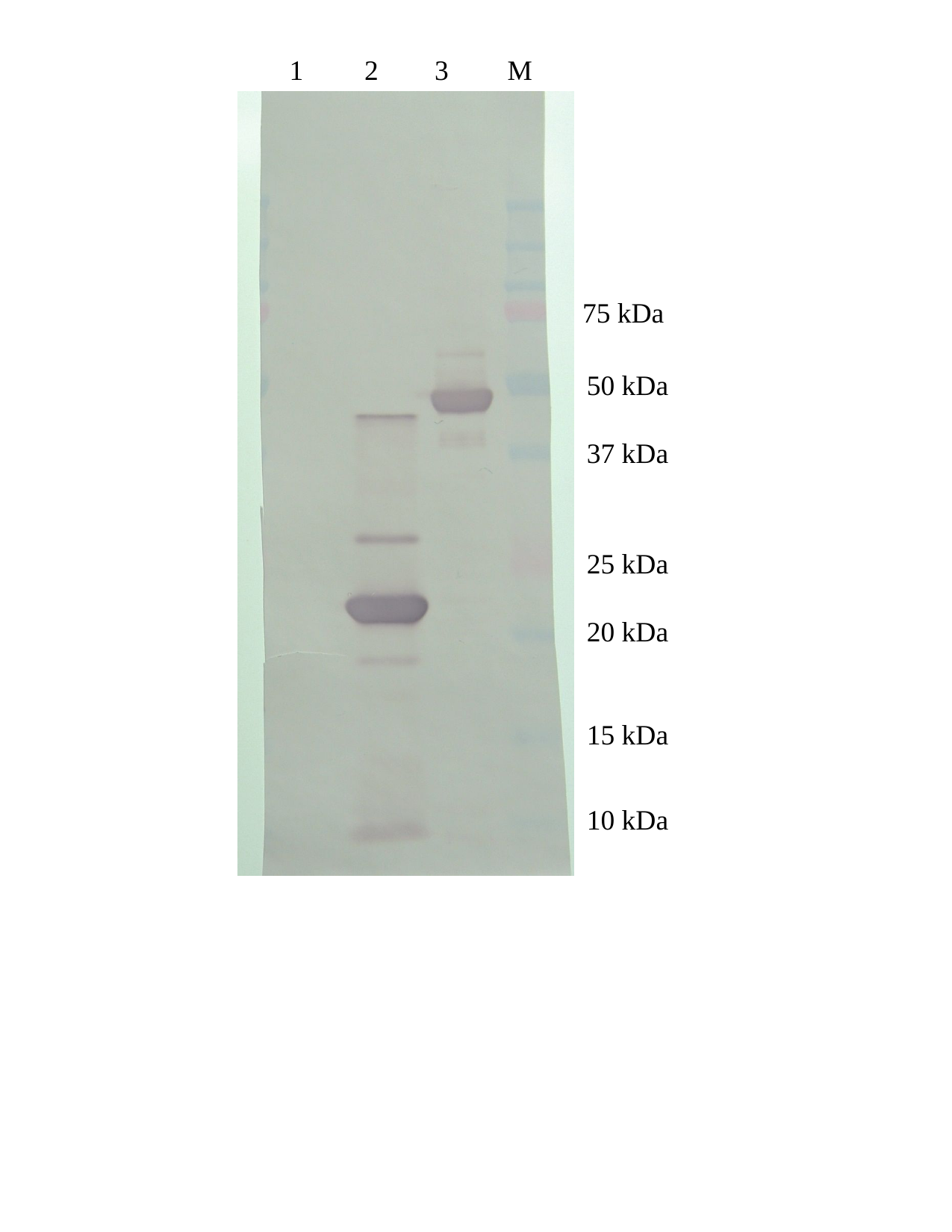

1
 2
3
M
75 kDa
50 kDa
37 kDa
25 kDa
20 kDa
15 kDa
10 kDa

Supplement: S3 Fig — The molecular weight marker (Bio-Rad Precision Plus Protein™ Standards), with the molecular masses of the individual standards, is shown in Lane M. (PPTX) [file pone.0230138.s004.pptx]

## Slide 1
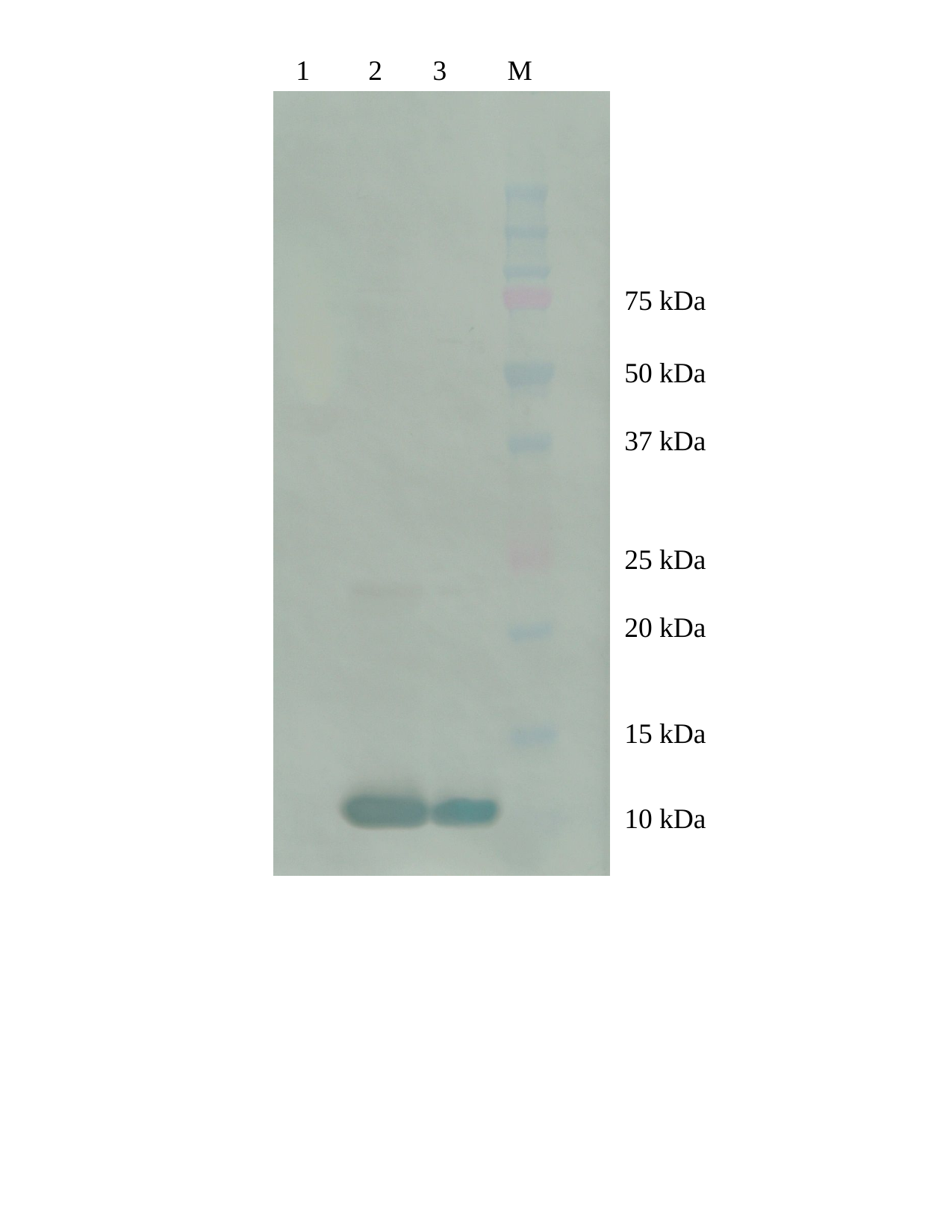

1
 2
3
M
75 kDa
50 kDa
37 kDa
25 kDa
20 kDa
15 kDa
10 kDa

Supplement: S4 Fig — The molecular weight marker (Bio-Rad Precision Plus Protein™ Standards), with the molecular masses of the individual standards, is shown in Lane M. (PPTX) [file pone.0230138.s005.pptx]
